# Beyond the Scope: The Hidden Curriculum in GI Fellowship and Its Implication for Change

**DOI:** 10.1007/s10620-025-09358-4

**Published:** 2025-08-27

**Authors:** Nicholas Noverati, Ananya Venkatesh, Gretchen Diemer, Aaron Martin

**Affiliations:** 1 Division of Gastroenterology and Hepatology, Department of Medicine, Thomas Jefferson University Hospital, 132 S 10th St Ste 480 Main, Philadelphia, PA 19107 USA; 2 Department of Medicine, Thomas Jefferson University Hospital, Philadelphia, PA USA; 3 Lewis Katz School of Medicine at Temple University, Philadelphia, PA USA

**Keywords:** Gastroenterology fellowship, Hidden curriculum, Training, Qualitative research

## Abstract

**Background:**

The hidden curriculum exists beyond formal and informal curricula and impacts a learner’s experience as something learned that is not intentionally taught. It is discussed extensively in undergraduate medical education literature, but less understood in graduate medical education, especially in a procedurally focused subspecialty training such as gastroenterology (GI) fellowship.

**Aims:**

To better understand examples of the hidden curriculum in GI fellowship, from the perspective of fellows, to improve the overall educational experience.

**Methods:**

A qualitative analysis of semi-structured virtual interviews using grounded theory was performed.

**Results:**

Twelve fellows of six different programs participated and revealed four main categories of themes: Hierarchies and Training Dynamics, Endoscopic and Procedural Training, Professional Aspirations and Career Perspectives, Patient Care, and Challenges.

**Conclusion:**

This study highlights areas of the GI fellowship learning experience that can be classified as part of the hidden curriculum and informs areas for further improvement: fostering a more collaborative learning environment, improving endoscopic training through formalized curricula and instructor professional development, addressing stereotypes in career decision-making, and modeling positive patient communication behaviors.

## Introduction

Becoming a fellow in Gastroenterology (GI) is a unique time in graduate medical education, as it not only affords the opportunity to attune clinical knowledge to that of a specialty of internal medicine but also introduces learning highly specific procedural skills for the first time. It is not uncommon that many new GI fellows have never operated an endoscope prior to starting fellowship. As fellowship includes adult learners who yearn to focus on that which is most relevant to them [1], it is important to consider how experiences can be optimized to acknowledge this, fostering the development of well-rounded, competent future gastroenterologists with both clinical and procedural expertise.

In 1998, Frederic Hafferty introduced a concept that stands outside of the formal and informal curriculum, known as the hidden curriculum, as a way to challenge the “traditional notion that changes to medical education are most appropriately made at the level of the curriculum” [2]. He specifically defined it as “a set of influences that function at the level of organizational structure and culture” [2].

Hafferty stresses, through this concept, the importance of the overall learning environment. Though we can attempt to accomplish learning objectives formally and even informally, the hidden curriculum begs the question: what is taught that is not explicitly intended? In this study, the hidden curriculum in GI fellowship is explored from the perspective of fellows in training, with the hope of discovering ways to improve upon the educational experience of fellows as adult learners. This has not been explored extensively in the current literature, which focuses more on the hidden curriculum of undergraduate medical education, especially as it pertains to its effect on professional development [3–5].

## Methods

**Fig. 1:**
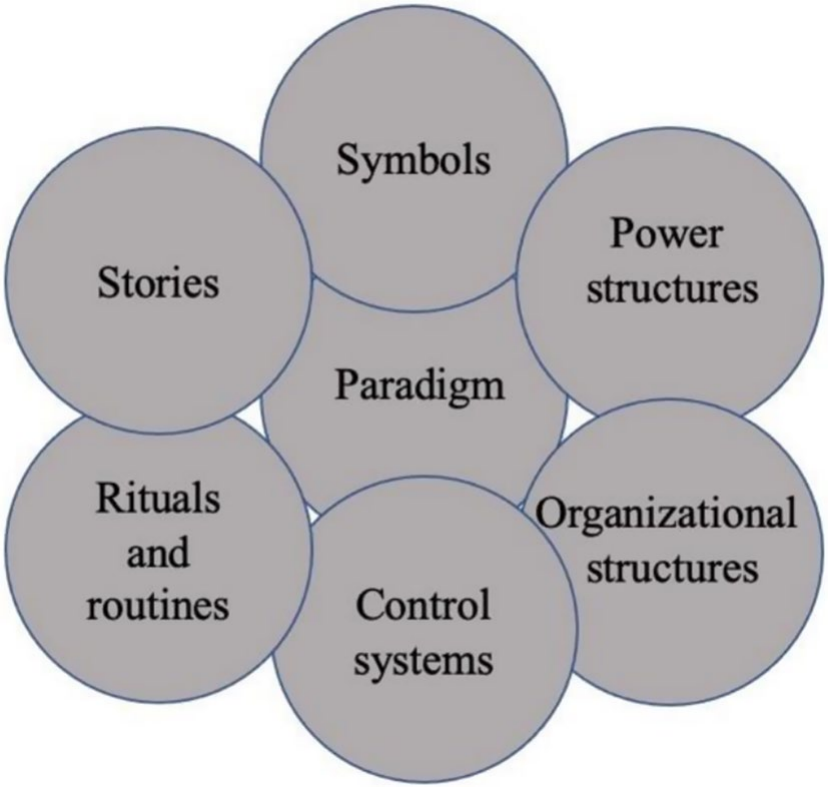
Cultural Web Model^6^

A qualitative approach was taken to uncover aspects of the hidden curriculum from the perspective of fellows. A cultural web model was adapted as a conceptual framework in this study [6]. This model was adapted from a business context and has the components of symbols, stories, paradigms, power structures, rituals and routines, control systems, and organizational structures and was found to be successful in revealing the hidden curriculum in medical education (see Fig. 1) [6]. The model was directly applied to the process of writing questions that fit each component of the web, to help guide semi-structured interviews with fellows currently enrolled in local GI/Hepatology programs.

Grounded theory methodology was adopted to analyze interview data, operating under the assumption that participants’ narratives would reveal elements of the hidden curriculum embedded within the underlying cultural web of their training programs.

Twelve fellows were interviewed using a non-probability sampling approach, which included convenience and snowball sampling; some participants were first identified through professional connections of author N.N. and others were referred by study participants (e.g., a participant’s co-fellow). Emails were sent through program contacts to ask for participation in an interview-based project.

Interviews were conducted virtually using the Zoom platform (https://zoom.com), in the year 2024, and typically lasted 30–45 min. Audio recording of the interviews was then transcribed using Otter.AI web software (https://otter.ai). The resulting transcripts were subsequently reviewed and proofread by author N.N. for accuracy.

Authors A.V. and G.D. were recruited by N.N. given their previous experience with qualitative research methods. A.V. and N.N. conducted qualitative analysis given their experience and expertise.

Transcribed and proofread interview documents were sent to both N.N. and A.V. for independent coding using Taguette, an open-source web-based text tagging tool (https:/www.taguette.org). Each author independently identified emerging themes from the coded data, employing an iterative approach that involved reviewing codes after two to three sessions, comparing them to prior sessions, and assessing whether data saturation had been reached. This was achieved at the end of twelve interviews, as no new substantial information emerged. The authors then met to review their independent analyses, finding their identified themes to be nearly identical. Through close collaboration, they refined the wording of the final themes and selected representative quotes. The finalized document of themes and quotes was subsequently shared with additional authors, G.D. and A.M., for review and external validation. To further ensure accuracy, findings were informally discussed with three participating fellows, who confirmed alignment with their experiences.

This study was reviewed by the Institutional Review Board (IRB) and was determined to be exempt by the Hooper Board #153 due to being categorized as educational research.

## Results

Twelve fellows, representing six different programs in two different geographic states in the U.S.A., participated (see Table 1). As labeled in Table 1, some participants were from programs deemed “academic,” which include faculty labeled clinical educators and typically include affiliation with medical schools and residency training programs. “Community-academic” programs typically include faculty that may or may not be assigned teaching roles. “Community” programs do not have an associated medical school or residency program. Three were post-graduate year (PGY)-4, three were PGY-5, and six were PGY-6. Seven were female and the average age was 31 years old.

**Table 1:** Fellow participant basic demographics and other characteristics

Fellow	Sex	Age	PGY	State of Fellowship	Fellowship Program Type	Job Interest
A	F	32	6	PA	Academic	Academics
B	F	30	5	PA	Academic	Undecided
C	F	29	4	NJ	Academic	Undecided
D	M	35	6	NJ	Academic	Academics
E	F	30	5	PA	Academic	Transplant Hepatology
F	M	30	5	PA	Academic-Community	Undecided
G	F	29	4	PA	Academic	Academics
H	F	33	6	PA	Academic-Community	Academic/community
I	M	32	6	PA	Academic	Academic
J	M	30	4	PA	Academic-Community	Undecided
K	F	32	6	PA	Academic-Community	Academic/community
L	M	31	6	NJ	Community	Private Practice

Qualitative analysis revealed seven themes within four broad categories: Hierarchies and Training Dynamics, Endoscopic and Procedural Training, Professional Aspirations and Career Perspectives, and Patient Care and Challenges (see Table 2).

**Table 2:** Categories, themes, and what aspect of the cultural web they fit into

Category	Themes	Aspect of Cultural Web
Hierarchies and Training Dynamics	There is a general sense of collegiality, but the hierarchy of medical training is still observed	Paradigm, Power Structures
	Fellows learn to conform to norms of their program	Paradigm, Power Structures
Endoscopic and Procedural Training	Learners adopt the view that endoscopic education is largely self-directed	Rituals and Routines
	Procedural planning is fellow-directed and requires complex skill	Rituals and Routines, Control Systems
Professional Aspirations and Career Perspectives	Private practice is viewed as more procedure and revenue driven with less work-life balance, whereas academic medicine is viewed more favorably especially among those training at academic programs	Stories
	Subspecialties in the field are often viewed through the lens of a stereotype	Symbols
Patient Care and Challenges	Fellows learn to identify “difficult” patients that will require more time, energy and patience. “Functional” patients usually seen as fitting this description	Control Systems

### Category 1: Hierarchies and Training Dynamics

#### Theme 1

There is a general sense of collegiality, but the hierarchy of medical training is still observed.

Fellows commented often on feeling a heightened sense of collegiality when compared to their experience in residency, as well as a generally positive interaction between members of the department. As an extension of this, they are more commonly introduced as colleagues by their attending. Despite this, there were clear examples of still feeling their place within an overarching hierarchy. For example, older, more traditional attendings were more likely to be dressed professionally in white coats, and fellows struggled more with calling them by their first name, even if invited to do so: “*it’s kind of hard to view them as a colleague when you’re so many years behind them*” (L), “*the older attendings are the ones who are dressed up*” (C), *“he will be like call me X, but he’s the only person in the entire institution who I still cannot call X… I have to call him Dr. X and I think it’s because he’s at the same level as all the other attendings who I call doctor”* (K).

#### Theme 2

Fellows learn to conform to norms of their program.

When fellows were asked to reflect on ways in which they have felt pressure to conform to a norm at their program, many reflected on learning to perform beyond formal requirements. Fellows at community programs were more likely to stress the importance placed on doing procedures while those in academic programs stressed research and scholarly pursuits: “*I think that would be the hope of the program, is that we do more research*” (A), “*you still have this kind of looming, like, this is your expectation and they give us protected time to do it*” (I), “*the culture at our program was, if you’re not done with notes and seeing patients before, you know, 7:30 AM, you’re not going to scope*” (L).

### Category 2: Endoscopic and Procedural Training

#### Theme 3

Learners adopt the view that endoscopic education is largely self-directed.

Fellows stated plainly that formal endoscopic curriculum is lacking. As a result, they are often introduced to the practice of scoping on their first day without any prior or graduated exposure: “*they kind of throw us in there, as I’m sure everyone is thrown in there… we don’t have a curriculum*,” (H) “*day one on consults and my attending has me scope and I’m like, holding a scope and just kind of putzing around the stomach*” (A). As a result, they stress the adapted need for self-directed learning, “*I just watched YouTube video after YouTube video and would go and play with the scopes and understand the dials*” (H). Fellows curate their experiences through repeated exposure to teachers of varying quality in order to develop their own set of skills, stating “*you need to get a feel…it’s kind of just trial and error, trial and error…I’ve only had two attendings that have actually been very good at walking you through it*” (D). Some mentioned eventually adopting the philosophy that this may be the nature of learning endoscopy “*everybody does it completely differently and there is no formal teaching and maybe that’s just like the nature of scoping*” (E).

#### Theme 4

Procedural planning is fellow directed and requires complex skill.

Fellows note the expectation that they are to manage the coordination of completing inpatient procedures, and there is a certain skill set required to do so, which includes learning how to navigate working with varying members of the endoscopy staff: “*the interaction and the kind of orchestra of doing procedures and interacting with other members of the team…that’s not something I had experience with as a resident*” (A), “*when you first start you’re a newbie. You’re a rookie. So like, you almost have to prove yourself. If you’re trying to add on a case, you have to prove to them*” (K). Careful planning, adherence to schedules, and identifying case priority were examples of specific skills: “*on top of all the consults you have to see, all the scopes you have to do, you have to be refreshing your phone every five minutes while you’re rounding to see if that repeat CBC came back*” (E). Understanding the limitations of endoscopy staffing, anesthesia’s pre-procedural requirements, and patient preparedness also aid in successfully completing a procedure: “*sometimes they’ll block certain cases because it’s too late, even though it’s like, I don’t know, 3:30 PM and we technically have the room until 5*” (B), “*I would say the biggest barrier is staffing, honestly*” (J), “*we have a very good relationship with anesthesia, but they are sometimes a little bit tricky because they kind of dictate whether or not we’re doing procedures*” (H).

### Category 3: Professional Aspirations and Career Perspectives

#### Theme 5

Private practice is viewed as more procedure and revenue driven, with less work-life balance, whereas academic medicine is viewed more favorably, especially among those training at academic programs.

As fellows learn about job opportunities after training, they reflect on what they are commonly told about jobs. Those training at academic programs are more often told about what academic practice is like and how it offers more work-life balance compared to private practice or private equity, which are seen as valuing reimbursement driven by procedural volume: “*private equity…when you’re speaking with our attendings… it has a little bit of a negative connotation to it, but I think that everyone sort of recognizes that this is coming from somebody that’s working currently in the academic role*” (F), “*the biggest thing I kept hearing was at the end of the day, you are just a number that’s going to create money for them, and they’re not looking at what your quality of life is as a physician*” (K). Fellows at community programs had a more balanced view of both perspectives.

#### Theme 6

Subspecialties in the field are often viewed through the lens of a stereotype.

When fellows were asked to reflect on stories that they have heard about subspecialties of GI, they stated specific examples. Advanced GI was viewed in the frame of a surgeon’s mentality and personality quirks: “*advanced attendings are like super intense,*” (I), and “*people who end up choosing advanced, it’s like they have a much more like surgeon mentality*” (K). Inflammatory bowel disease (IBD) subspecialists were often seen as cerebral, “*very meticulous, calm and patient and willing to listen to their patients talk for hours*” (E). Hepatology was regarded similarly. General GI was seen as procedure heavy with less complexity (unless practicing in academics): “*gen GI just want to do colonoscopies all day and don’t want to use their brains*” (I). In general, doing a super-fellowship was often viewed as martyr: “*the biggest stereotype I see is like, oh my, why would you want to prolong your training anymore*” (K) and “*we have one guy who’s trying to do a transplant year and everyone’s like ‘he’s doing the noble thing*’” (C).

### Category 4: Patient Care and Challenges

#### Theme 7

Fellows learn to identify “difficult” patients that will require more time, energy, and patience. “Functional” patients are usually seen as fitting this description.

Most fellows acknowledge that there will be some patient encounters that are more difficult than others and have chosen to model good attributes of their attendings’ behavior, which often involves a creative approach. Examples included “*I’ve seen it go poorly before where an attending will either rush them or kind of try to interject… [but] I have learned that you just have to let them talk and get it all out*” (E) and “*certainly there’s some attendings who I don’t want to say are dismissive, but just tend to try to limit the interactions appropriately*” (A). There are known stereotypes of patients who are predicted to be more difficult and time consuming, and they often hold diagnoses within disorders of gut brain interaction (DGBI) or motility disorders. Certain attitudes toward patients can be negative and are perpetuated over time: “*definitely certain stereotypes that patients can have…like your typical gastroparesis patients and…it’s like… oh my goodness, not this person again*” (C), “*the motility patients, the gastric stimulators….I mean, even some of the more difficult esophageal achalasia patients can be challenging*” (A), “*the second a young woman who has chronic abdominal pain comes in…like, everybody rolls their eyes, including me*” (B).

## Discussion

In the spirit of Hafferty’s attempt to uncover a different, “hidden” curriculum that includes learning not explicitly intended yet part of the environment that affects a learner, this qualitative analysis provides insight into what this might look like in GI fellowship training. Four categories of seven main themes were identified: Hierarchies and Training Dynamics, Endoscopic and Procedural Training, Professional Aspirations and Career Perspectives, and Patient Care and Challenges.

In terms of hierarchies and training dynamics, despite more often feeling like a colleague to their attendings, there continues to be a learned sense of “place” within a hierarchy. As previously discussed in the literature, hierarchies in medical training can be helpful as they allow trainees to feel supported in the development of clinical skills [7]. If left unattended, power differentials introduced through a hierarchy can become repressive and lead to those of lower “rank” to feel uncomfortable raising a myriad of concerns, from patient care to problems in the workplace [7]. Though fellows were more often to be introduced as colleagues, they often did not feel comfortable with something as simple as addressing attendings by their first name, even if invited to do so. This could, perhaps, be highlighting a disconnect between an ideally “functional” and “dysfunctional” hierarchy, and a possible opportunity to further encourage collaboration between fellows and faculty to foster a more inclusive learning environment.

Fellows also commented on needing to direct their own learning of endoscopic skills. This stresses the need for a more formalized curriculum or, at the least, a structured curricula that combines self-directed learning with intentional faculty guidance. Programs might benefit from explicitly acknowledging the role of repeated exposure to procedures over time, leading to improved competence, but while further developing instructors’ ability to both assess skills and translate their own competence onto their learners (also termed “conscious competence”) [8]. Though this finding clearly highlights a deficit in educational programming, it also highlights that the hidden curriculum of “expecting” to self-direct learning (without explicitly being told to) is serving as a substitute for more formalized curricula.

Qualitative analysis also revealed that fellows often have to learn to navigate completing inpatient procedures through balancing complex non-clinical skills. This might be content to intentionally include in curricula, such as during fellowship orientation or otherwise delivered to novice learners.

Career choices in the field of GI and Hepatology were often seen through the lens of learned stereotypes. This underscores the importance of supporting fellows in pursuing subspecialties that interest them, rather than perpetuating preconceived notions.

Interview data also revealed that fellows learn to manage difficult patients by emulating positively displayed behaviors, but easily identify specific populations known to be “difficult.” This is an example of the hidden curriculum, as fellows learn through their experiences which patients are seen as “difficult” and which are not, without explicitly being taught as such. Faculty should remain mindful of how their attitudes toward challenging patients may shape fellows’ perceptions and approach to future patient care, especially those with specific diagnoses such as DGBI.

Though there are undoubtedly positive experiences that also constitute the hidden curriculum, most themes explored here can subjectively be viewed as negative. However, this is likely due to the phrasing of the questions asked, which were largely shaped by the objective of this study to explore how improvements to fellowship training can be made. Further, as previously published, the hidden curriculum typically takes on a “negative” connotation just by nature of the term [5]. Even other surgical subspecialties have published on trainees’ perceived toxic stereotypes, similar to those identified here for GI subspecialties [9]. Contrarily, one clear positive example of the hidden curriculum, which has been more extensively discussed, is that of role models transmitting and reinforcing professional behaviors to medical trainees [3, 10]. This is similar to theme 7, which involves learning skills to manage difficult patients.

This study is limited by its use of non-probability sampling methods, which may introduce sampling and selection bias. To mitigate this, we included fellows from multiple different programs to enhance the generalizability of our findings. However, as the programs were located in relatively close geographic proximity, the findings may also reflect regional cultural norms. As the hidden curriculum is influenced by culture, it is possible that findings might be different in different geographic locations. Although this study included a small sample size, the qualitative analysis performed revealed that saturation in responses was reached. As previously discussed in the literature, this can be accomplished with a narrow range of participants of a more homogeneous phenotype [11]. Thus, though there may be regional cultural norms in our study population, it also allowed for a smaller sample size to accomplish saturation. Future research might combine the use of quantitative and qualitative methodologies and include groups of fellows in different locations to allow for a more comprehensive understanding.

Overall, the findings here point to several key areas for improvement in the GI fellowship learning experience. By addressing these aspects of the hidden curriculum, fellowship programs can better support learners’ professional, procedural, and interpersonal growth, better supporting their adult learning needs [1].

## Data Availability

No datasets were generated or analyzed during the current study.
